# A study of the relationship between human infection with avian influenza a (H5N6) and environmental avian influenza viruses in Fujian, China

**DOI:** 10.1186/s12879-019-4145-6

**Published:** 2019-09-02

**Authors:** Ping Chen, Jian-Feng Xie, Qi Lin, Lin Zhao, Yan-Hua Zhang, Hong-Bin Chen, Yu-Wei Weng, Zheng Huang, Kui-Cheng Zheng

**Affiliations:** 1 College of Public Health, Fujian Medical University, No. 88, Jiaotong Road, Taijiang District, Fuzhou, 350000 China; 2 Fujian Center for Disease Control and Prevention, Fujian Provincial Key Laboratory of Zoonosis Research, Fuzhou, 350001 China

**Keywords:** Avian influenza a (H5N6) virus, Environmental contamination, Phylogenetic analysis

## Abstract

**Background:**

Avian influenza A (H5N6) virus poses a great threat to the human health since it is capable to cross the species barrier and infect humans. Although human infections are believed to largely originate from poultry contaminations, the transmissibility is unclear and only limited information was available on poultry environment contaminations, especially in Fujian Province.

**Methods:**

A total of 4901 environmental samples were collected and tested for Avian Influenza Virus (AIV) from six cities in Fujian Province through the Fujian Influenza Surveillance System from 2013 to 2017. Two patient-related samples were taken from Fujian’s first confirmed H5N6 human case and his backyard chicken feces in 2017. *Chi-*square test or Fisher’s exact probability test was used to compare the AIV and the viral subtype positive rates among samples from different Surveillance cities, surveillance sites, sample types, and seasons. Phylogenetic tree analysis and molecular analysis were conducted to track the viral transmission route of the human infection and to map out the evolutions of H5N6 in Fujian.

**Results:**

The overall positive rate of the H5 subtype AIVs was 4.24% (208/4903). There were distinctive differences (*p* < 0.05) in the positive rates in samples from different cities, sample sites, sample types and seasons. The viruses from the patient and his backyard chicken feces shared high homologies (99.9–100%) in all the eight gene segments. Phylogenetic trees also showed that these two H5N6 viruses were closely related to each other, and were classified into the same genetic clade 2.3.4.4 with another six H5N6 isolates from the environmental samples. The patient’s H5N6 virus carried genes from H6N6, H5N8 and H5N6 viruses originated from different areas. The R294K or N294S substitution was not detected in the neuraminidase (NA). The S31 N substitution in the matrix2 (M2) gene was detected but only in one strain from the environmental samples.

**Conclusions:**

The H5 subtype of AIVs has started circulating in the poultry environments in Fujian Province. The patient’s viral strain originated from the chicken feces in his backyard. Genetic reassortment in H5N6 viruses in Fujian Province was indicated. The H5N6 viruses currently circulating in Fujian Province were still commonly sensitive to Oseltamivir and Zanamivir, but the resistance against Amantadine has emerged.

**Electronic supplementary material:**

The online version of this article (10.1186/s12879-019-4145-6) contains supplementary material, which is available to authorized users.

## Background

China’s first H5N6 strain was isolated in March 2014 from a domestic duck in Guangdong Province [1]. Since then, the H5 subtype has gradually become the main cause of the sporadic AIV infections in poultry in southern China [2, 3].

Wild migratory birds and waterfowls are often considered as natural hosts of AIVs. Through their migration, the viruses are transmitted globally [4, 5]. The A(H5) hemagglutinin(HA) gene from the highly pathogenic avian influenza (HPAI) A(H5N1) viruses, which were derived from the A/goose/Guangdong/1/96 (gs/GD/96) H5 HA lineage, has continued to rapidly evolve since the most recent update of the H5clade nomenclature by the WHO/OIE/FAO H5N1 Evolution Working Group [6, 7]. A new HA clade, which has included H5N6, H5N1, H5N8, H5N2 and other subtypes of AIVs since 2014, was identified and named as clade 2.3.4.4 [6, 8, 9]. So far, only the H5N6 viral subtype in the clade 2.3.4.4 has been reported to cross the species barrier and infect humans [10]. In April 2014, the first confirmed human case of avian influenza A (H5N6) virus infection was reported in Sichuan Province, China [11]. As of mid-2017, a total of 17 human H5N6 infections and 7 deaths were reported in China [12]. In December 2017, Fujian Center for Disease Control and Prevention (FJCDC) identified Fujian’s first and China’s nineteenth human infection of H5N6 virus [13].

Epidemiological investigations have confirmed that most cases of H5N6 human infections had contact with infected poultry [14–16]. Live poultry markets (LPMs) and backyard poultry flocks (BPFs) are two major risk factors for human infections [17, 18]. It had also been noticed that in many provinces, human infections of AIVs decreased gradually after closing LPMs [19].

Therefore, in order to reduce the risk of human H5N6 infection and the impacts of viral circulation in poultry, and also to trace the origin of the human H5N6 virus, we collected and analyzed the samples from poultry environments and the human H5N6 virus in Fujian Province, China.

## Methods

### Data collection and analysis

Data for this study were collected through the Fujian Influenza Surveillance System, which has been collecting at least 40 environmental samples monthly from locations such as LPMs, poultry farms, households and slaughter factories in Fuzhou, Xiamen, Quanzhou, Sanming, Nanping and Zhangzhou cities since 2013. Sample types includes surface wipe samples of poultry cage and slaughter or placing board, poultry fecal samples, poultry cleaning sewage samples and poultry drinking water samples. Samples were kept under 4 °C to 8 °C and sent to FJCDC within 24 h for viral detections. Surveillance data from 2013 to 2017 were retrieved and analyzed using SPSS 20.0. The differences of positive detection rates of avian influenza A and the H5 subtype in different network labs, surveillance sites, sample types, and seasons were tested by *chi*-square test or Fisher’s exact probability test.

### Virus isolation and sequencing

The two case-related samples were injected into 9-to-10-day-old specific pathogen-free (SPF) embryonated chicken eggs and inoculated at 37 °C for 72 h in a biosafety level 3 laboratory. Hemagglutination assay and hemagglutination inhibition assay of the allantonic fluids were sequentially used for subtype identifications.

Viral RNA was amplified using Qiagen One-Step RT-PCR Kit (Qiagen, GmbH) with 32 pairs of primers (Additional file 1: Table S1) under the following conditions: 95 °C for 15 min, 50 °C for 30 min, 94 °C for 2 min, followed by 35 cycles of 94 °C for 30 s, 56 °C for 30 s and 72 °C for 1 min, and finally at 72 °C for 10 min. PCR products were purified with QIAquick Gel Extraction Kit (Qiagen, GmbH) and sequenced using ABI 3500 genetic analyzer (Life Technologies, Grand Island, NY, USA) following the manufacturer’s instructions.

Nucleotide sequencing was performed on an ABI 3500 genetic analyzer (Life Technologies, Grand Island, NY, USA) using the ABI BigDye Terminator v3.1 Cycle Sequencing Kit (Life Technologies) following the manufacturer’s instructions. The sequencing primers were M13F (5′–TGTAAAACGACGGCCAGT–3′) and M13R (5′–CAGGAAACAGCTATG ACC–3′). Sequences were assembled using the SeqMan program of the Lasergene Package (DNASTAR, Inc.; Madison, WI, USA). The genetic sequences of both strains were submitted to the Global Initiative on Sharing Avian Influenza Data (GISAID) (http://platform.gisaid.org/). Full genetic sequences of all six Fujian H5N6 strains from 2016 were retrieved from the China Influenza Virus Genetic Sequence Database and the GISAID EpiFlu Database (isolate ID numbers are presented in Additional file 2: Table S2).

### Phylogenetic analysis

The DNAMAN program (version 6.0) was used for the analysis and alignment of the sequencing data. The phylogenetic trees were performed in MEGA version 6 (The Biodesign Institute, Tempe, AZ, USA) using maximum-likelihood method based on the Tamura–Nei model with 1000 bootstrap replicates [20]. Several typical sequences of other viral subtypes from Asia, especially from the Yangtze River Delta and the Pearl River Delta in China, were also obtained from GISAID and used for constructing phylogenetic trees. (GISAID isolated ID numbers are listed in Additional file 3). The reference sequences were as follows: A/Sichuan/26221/2014 (H5N6), A/Guangzhou/39715/2014 (H5N6), A/Guangdong/99710/2014 (H5N6), A/Changsha/1/2014 (H5N6), A/Guangdong/ZQ874/2015 (H5N6), A/Shenzhen/1/2015 (H5N6), A/Anhui/33163/2016 (H5N6), and A/Hubei/29578/2016 (H5N6). GISAID isolate ID of the viruses above were listed in Additional file 2: Table S2.

## Results

### Statistical analysis of surveillance samples

The positive detection rates of influenza A virus and the H5 subtype were 31.92% (1565/4903) and 4.24% (208/4903), respectively. There were statistical differences in the positive rates of influenza A virus and the H5 subtype in different cities, sample sites, samples types and seasons (*p* < 0.05). The highest positive rates of the H5 subtype were observed in Sanming city (9.83%), LPMs (5.26%), and the wipe samples of poultry chopping board (7.66%). The positive rates of H5 subtype were at peak in winter and spring (Table 1). The subtypes in the other positive samples were shown in Additional file 1: TableS1.

**Table 1:** Surveillance results of environmental AIVs in Fujian Province, during 2013–2017

			Influenza A virus			H5 subtype		
		Number of samples	Number of positive (%)	*P*-value	*χ* ^2^	Number of positive (%)	*P*-value	*χ* ^2^
Surveillance city	Fuzhou	819	157 (19.17)	<0.001	540.835	7 (0.85)	<0.001	101.033
	Xiamen	1060	594 (56.04)			63 (5.94)		
	Quanzhou	1234	369 (29.90)			51 (4.13)		
	Zhangzhou	589	33 (5.60)			1 (0.17)		
	Sanming	600	219 (36.50)			59 (9.83)		
	Nanping	601	193 (32.11)			27 (4.49)		
Sample site	LPMs	3785	1500 (39.63)	<0.001*		199 (5.26)	<0.001*	
	Poultry farms	275	1 (0.36)			0 (0.00)		
	Poultry households	701	48 (6.85)			4 (0.57)		
	Poultry slaughter factories	133	16 (12.03)			5 (3.76)		
	Others	9	0 (0.00)			0 (0.00)		
Sample	Fresh fecal	1696	477 (28.13)	<0.001*		55 (3.24)	<0.001*	
	Cage surface	1245	401 (32.21)			35 (2.81)		
	Poultry drinking water	788	252 (31.98)			39 (4.95)		
	Cleaning poultry sewage	635	249 (39.21)			38 (5.98)		
	Poultry chopping board surface	535	184 (34.39)			41 (7.66)		
	Others	4	2 (50.00)			0 (0.00)		
Season	Spring	1448	555 (38.33)	<0.001	90.916	78 (5.39)	<0.001	48.174
	Summer	1060	284 (26.79)			20 (1.89)		
	Autumn	975	218 (22.36)			19 (1.95)		
	Winter	1420	508 (35.77)			91 (6.41)		
	Total	4903	1565 (31.92)			208 (4.24)		

*P value counted by Fisher’s exact probability

### Phylogenetic analysis of the H5N6 viruses

Viral isolates from the confirmed case and his courtyard chicken feces shared 99.9 to 100.0% nucleotide sequence identities in eight gene segments (Table 2). This result revealed that the human case was infected through the close contact with his contaminated courtyard poultry environment.

**Table 2:** Homology analysis between human H5N6 virus and H5N6 viruses from environments shed by infected poultry

Isolates	A/Fujian-Sanyuan/21099/2017 (Identities %)							
	PB2	PB1	PA	HA	NP	NA	MP	NS
A/Environment/Fujian/05324/2016	88.4	88.1	91.6	93.9	98.4	96.3	90.5	90.4
A/Environment/Fujian/05326/2016	85.1	90.1	91.6	94.1	97.2	98.2	90.1	90.4
A/Environment/Fujian/09991/2016	85.4	87.2	91.9	94.1	98.0	98.5	90.2	91.2
A/Environment/Fujian/28681/2016	88.8	88.0	87.8	94.0	91.8	95.7	87.6	87.7
A/Environment/Fujian/28686/2016	85.4	87.2	91.9	94.0	98.1	98.5	90.2	91.2
A/Environment/Fujian/52356/2016	88.5	88.1	91.5	93.5	97.8	95.9	90.3	89.8
A/Environment/Fujiansanyuan/08/2017	100.0	100.0	99.9	100.0	99.9	99.9	100	100.0

**Fig. 1:**
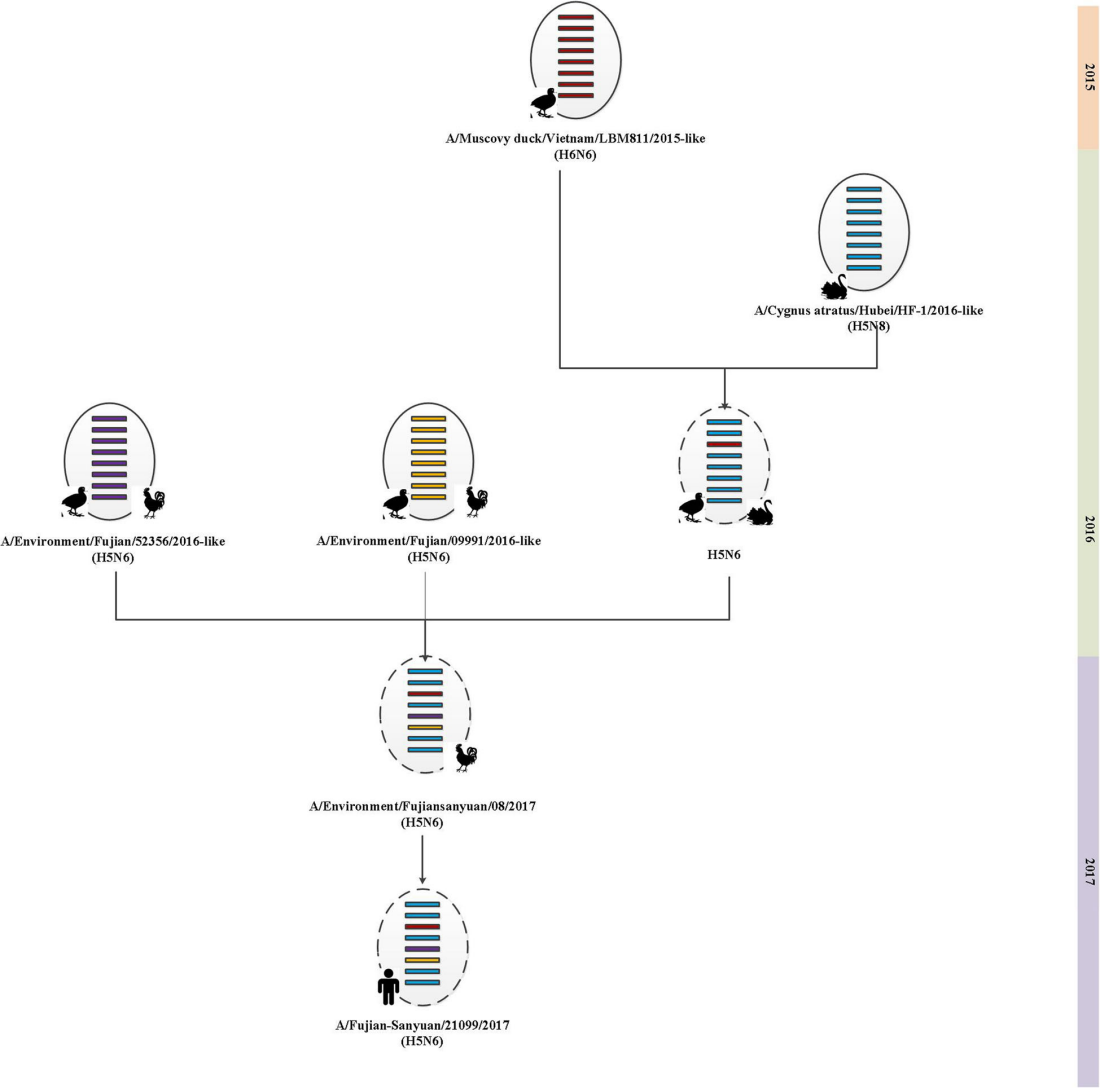
Hypothetical lineage for the origin of the human H5N6 virus in Fujian Province, China. The ovals represent virus particles and horizontal bars represent the eight gene segments (from top to bottom: PB2, PB1, PA, HA, NP, NA, MP, NA). The broken oval represent a genetic reassortment virus

Phylogenetic tree analysis also confirmed that the eight gene segments of the patient strain (A/Fujian-Sanyuan/21099/2017) were closely related to the courtyard chicken feces strain (A/environment/fujiansanyuan/08/2017). Their PA genes were both clustered into the H_X_N6 viruses and were the closest to the H6N6 strain of A/Muscovy duck/Vietnam/LBM 811/2015. Their polymerase basic 2 (PB2), polymerase basic 1 (PB1), HA, matrix protein (MP) and nonstructural (NS) genes appeared to be derived from an H5N8 virus isolated from a black swan in Hubei Province, China (A/Cygnus atratus/Hubei/HF-1/2016). Their NA and nucleoprotein (NP) gene segments were in the same cluster as the H5N6 viruses that were circulating in Fujian Province during 2016 (Additional file 1 and Fig. 1). These findings demonstrated that A/Fujian-Sanyuan/21099/2017, a triple reassortant virus, had reasserted from H6N6, H5N8, and H5N6 viruses. According to the WHO nomenclature system [21], the human H5N6 virus and the other 7 environmental H5N6 isolates from this study were clustered into clade 2.3.4.4, same as the other human H5N6 strains from different provinces. However, the human H5N6 strain in Fujian had a genetic distance from the other human strains. In addition, the human H5N6 strain in this study and the human H5N1 strain isolated in 2005 in Fujian Province both belonged to clade 2.3.4, however in two different sub-branches. These results showed that the H5 subtype in Fujian Province has been constantly evolving, and the human H5N6 virus in Fujian Province may be a novel reassortant virus.

### Molecular characteristics of the H5N6 viruses

The key amino acid mutations had increased the viral mammalian transmissibility, virulence in mammals and antiviral resistance in the human strain and the other 7 environmental strains of H5N6 viruses (Table 3).The HA of all eight virus strains showed a multiple basic amino acid motif of RERRRKR↓GL, indicating high pathogenicity in poultry. However, the Q226L and G228S mutations that may enhance the risk of human infection were not identified at the 210-loop in the HA proteins in all eight virus strains. The previously reported deletion of the five amino acids at the position 69 to 73 in the stalk region of NA residue was not observed in the eight H5N6 viruses from Fujian. Neither R294K nor N294S substitution was detected in the NA genes of all the eight H5N6 viruses, indicating a general sensitivity to Oseltamivir and Zanamivir antiviral drugs. However, the S31 N substitution in the M2 gene was detected in one strain of A/Environment/Fujian/28681/2016. Other mutations that have been shown to increase virulence in mice, such as L89 V, G309D, T339K, R477G and I495V in PB2, P42S in NS1, N30D and T215A in M1, were also detected.

**Table 3:** Molecular analysis of H5N6 virus isolates in Fujian Province, China

Viral Protein	Mutations#	Virus^1^	Virus^2^	Virus^3^	Virus^4^	Virus^5^	Virus^6^	Virus^7^	Virus^8^	Function
HA	Q226L*	Q	Q	Q	Q	Q	Q	Q	Q	Increased α2,6-SA recognition [22]
	G228S*	G	G	G	G	G	G	G	G	Increased α2,6-SA recognition [22]
	Cleavage site	RERRR KR↓GL	RERRR KR↓GL	RERRR KR↓GL	RERRR KR↓GL	RERRR KR↓GL	RERRR KR↓GL	REKRR KR↓GL	RERRR KR↓GL	Enhanced pathogenic effects in poultry [23]
NA	69-73deletion	No	No	No	No	No	No	No	No	Increased virulence in mice [24]
	R294K/N294S	R	R	R	R	R	R	R	N	Oseltamivir and zanamivir resistance [25]
PB2	L89V	V	V	V	V	V	V	V	V	Enhanced pathogenicity in mice [26]
	G309D	D	D	D	D	D	D	D	D	Increased virulence and replication in mice [27]
	T339K	K	K	K	K	K	K	K	K	Increased virulence and replication in mice [27]
	R477G	G	G	G	G	G	G	G	G	Increased virulence and replication in mice [27]
	I495V	V	V	V	V	V	V	V	V	Increased virulence and replication in mice [27]
	E627K	E	E	E	E	E	E	E	E	Mammalian host adaptation [28]
	D701N	D	D	D	D	D	D	D	D	Enhanced transmission in guinea pigs [29]
PB1	H99Y	H	H	H	H	H	H	H	H	Enhanced H5 virus transmissible among ferrets [26]
	I368V	I	I	I	V	I	I	I	I	Enhanced H5 virus transmissible among ferrets [26]
NS1	P42S	S	S	S	S	S	S	S	S	Increased virulence in mice [30]
	D92E	E	E	E	D	E	E	D	D	Increased virulence in mice and pigs [27]
	218-230deletion	Yes	Yes	Yes	Yes	Yes	Yes	No	No	Lack of PDZ domain binding motif:decreased virulence in mice [31]
M1	N30D	D	D	D	D	D	D	D	D	Increased virulence in mice [32]
	T215A	A	A	A	A	A	A	A	A	Increased virulence in mice [32]
M2	S31N	S	S	S	N	S	S	S	S	Amantadine resistance [33]

## Discussion

Since the H5N1 virus was first identified in Guangdong Province, China, 1996, this high pathogenic H5 subtype of AIVs has been continually detected in wild birds and domestic poultry environments [34]. The H5N1 virus has gradually evolved into novel reassortant H5Nx viruses of different NA subtypes since 2008 [35]. In later years, the H5N6 virus has spread among poultry in the southern and western China [2]. Poultry outbreaks of H5N6 were recorded in China, Laos and Vietnam in 2014 [36]. In China, LPMs played a key role in amplifying and disseminating AIVs through poultry market chains, where the reassortment and the emergence of novel viruses often take place [15]. The positive rate of H5 subtype in LPMs in Fujian Province was 5.26%, which was significantly higher than the other sample sites. Our data also showed that, during 2013 to 2017, the H5 subtype of AIVs had been circulating in the 6 surveillance cities in Fujian Province, with peak positive detection rates in the winter and spring seasons. These seasonal peaks were consistent with the seasonal trend of human avian influenza A infections [37]. Attentions need to be paid to the prevention and control of avian influenza in areas with high positive rates of the H5 subtype in winters and springsand in the poultry environment, to reduce viral reassortments and therefore viral transmissibility in mammals.

The H5N6 viral isolates from the first human case in 2014 carried the same internal genes as the H5N1 virus identified in 2013 [3]. In the same year, Korea reported the H5N8 subtype of avian influenza virus [38], which subsequently spread across East Asia, North America and Europe [39]. The H5N8 and H5N6 both belong to clade 2.3.4.4 [40]. However, different from the H5N8 viruses, the circulation of H5N6 viruses is limited in China, Laos, and Vietnam [10]. In Fujian Province, the recently circulating H5N6 (clade 2.3.4.4) and the previously circulating H5N1 (clade 2.3.4) isolates in 2005 were classified into different clusters by the phylogenetic analysis of the HA genes, indicating a transition of the dominating virus in poultry circulation.

Influenza viruses are carried by migratory waterfowl and therefore spread globally along the avian flyways during migration [41]. H5N6 is also circulated among migratory birds [42]. During migration seasons, the risk of geographical spread of AIVs is elevated. The shallow lakes connected to the Yangtze River and the Pearl River have copious biological resources and serve as important stopover sites for large flocks of migratory birds. Fujian Province, located between the Yangtze River and the Pearl River, is an important stopover point along the East Asian-Australasian Flyway, inhabiting large and various flocks of globally migratory birds [43, 44]. Genetic reassortments and viral evolutions are constantly occurring in Fujian Province because of its geological location and its dense and scaled poultry industries. Phylogenetic analysis showed that the environmental H5N6 viruses were closely related to the H5N6, H5N8, and H6N6 viruses that have been circulating among poultry in China. It was therefore speculated that the viruses analyzed in this study originate from poultry, not wild birds. Furthermore, the human H5N6 strain in this study had genetic distance from the other human strains from different provinces in recent years. It was concluded that the human H5N6 strain in this study may be a novel reassortant virus.

The H5N6 viruses in this study were considered highly pathogenic in poultry due to the amino acids at the HA cleavage site. The affinities of the influenza virus to different sialyl-sugar structures are important determinants of rang and pathogenicity in the viral host [26]. The α-2,6 sialyl glycan binds with human influenza viruses, but the α-2,3 sialyl glycan binds with avian viruses [45]. The Q226L and G228S mutations in HA were reported to bind with the α-2,6 sialyl glycan receptors. In this study, we didn’t find these mutations that could increase viral adaption to human hosts. The 69–73 amino acids deletion in NA were observed to enhance virulence in mice [24], and may be associated with the viral adaptation and transmission in poultry [26, 46]. However this deletion was not observed in the H5N6 virus strains in our study. Based on the amino acid sequences of A/Fujian-Sanyuan/21099/2017, the human strain was still sensitive to Oseltamivir and Zanamivir. One of environmental H5N6 strains was resistant to Amantadine. Other virulence markers were found in PB2, PB1, NS1 and M1. However, these mutations were only studied in animals, and their effects on the virulence in human hosts require further research.

Our study has several strengths, including large environmental sample size, viral isolated from Fujian’s first confirmed H5N6 human case and his courtyard chicken feces. However, this study also has some limitations, for example, the signs and symptoms and mortality rates among poultry were not collected.

## Conclusions

The results of this study indicated that in Fujian Province, the clade 2.3.4.4 of the H5 subtype had become the main circulating AIVs in poultry environments. The patient with H5N6 infection was most likely to contract the virus from contaminated poultry environment. The human strain had genetic reassortment with H6N6, H5N8, and H5N6 viruses. It was still sensitive to Oseltamivir and Zanamivir. Although there has been no outbreak of human infection with H5N6 in Fujian Province, it is of great importance to continue and strengthen the surveillance of the H5Nx virus in poultry environment to monitor the spread and evolution of the virus.

## Additional files

Additional file 1: **Table S1.** Composition of subtypes of AIVs positive specimens in Fujian Province, during 2013–2017. H5 + H7: Both the H5 and H7 subtype influenza virus nucleic acids were detected in the same sample and other subtypes and so on. (DOCX 18 kb)

Additional file 2: **Table S2.** The GISAID isolate ID of H5N6 viruses. (DOCX 16 kb)

Additional file 3: Phylogenetic analysis of the H5N6 viruses isolated in Fujian Province. (In red Triangle) Viral strains of human infection with avian influenza A(H5N6) virus in Fujian Province. (In green Square) Viral strains of human H5N6 viruses. (In red Circle) Viral strains of H5N6 viruses isolated from environment sample in Fujian Province. (DOC 415 kb)

## Data Availability

The dataset used and analyzed in this study are stored in GISAID and FJ CDC. The datasets are available upon reasonable requests to the corresponding author.

## Abbreviations

AIV: Avian Influenza Virus
BPFs: Backyard poultry flocks
FJCDC: Fujian Center for Disease Control and Prevention
GISAID: Global Initiative on Sharing Avian Influenza Data
HA: Hemagglutinin
HPAI: Highly pathogenic avian influenza
LPMs: Live poultry markets
MP: Matrix protein
NA: Neuraminidase
NP: Nucleoprotein
NS: Nonstructural proteins
PA: Acidic polymerase
PB1: Basic polymerase 1
PB2: Basic polymerase 2
