# Supplementary material for: A study of the relationship between human infection with avian influenza a (H5N6) and environmental avian influenza viruses in Fujian, China
Source: BMC Infect Dis. 2019 Sep 2;19:762. doi: 10.1186/s12879-019-4145-6 (PMC6719373; doi:10.1186/s12879-019-4145-6)
Supplement: Supplementary file 1 — Table S1. Composition of subtypes of AIVs positive specimens in Fujian Province, during 2013–2017. H5 + H7: Both the H5 and H7 subtype influenza virus nucleic acids were detected in the same sample and other subtypes and so on. (DOCX 18 kb) [file 12879_2019_4145_MOESM1_ESM.docx]

|  |  | H5 | H7 | H9 | H5+H7 | H5+H9 | H7+H9 | H5+H7+H9 | A Unknown |
| --- | --- | --- | --- | --- | --- | --- | --- | --- | --- |
| Surveillance city | Fuzhou | 6 | 17 | 41 | 0 | 1 | 1 | 0 | 91 |
|  | Xiamen | 21 | 10 | 374 | 1 | 38 | 77 | 3 | 70 |
|  | Quanzhou | 45 | 163 | 146 | 0 | 4 | 7 | 2 | 2 |
|  | Zhangzhou | 0 | 3 | 23 | 0 | 0 | 5 | 1 | 1 |
|  | Sanming | 31 | 8 | 80 | 4 | 21 | 7 | 3 | 65 |
|  | Nanping | 27 | 5 | 104 | 0 | 0 | 17 | 0 | 40 |
| Sample site | LPMs | 122 | 197 | 728 | 5 | 64 | 111 | 8 | 265 |
|  | Poultry farms | 0 | 1 | 0 | 0 | 0 | 0 | 0 | 0 |
|  | Poultry households | 3 | 8 | 29 | 0 | 0 | 3 | 1 | 4 |
|  | Poultry slaughter factories | 5 | 0 | 11 | 0 | 0 | 0 | 0 | 0 |
|  | Others | 0 | 0 | 0 | 0 | 0 | 0 | 0 | 0 |
| Sample | Fresh fecal | 35 | 53 | 246 | 1 | 17 | 38 | 2 | 85 |
|  | Cage surface | 22 | 51 | 200 | 1 | 11 | 36 | 1 | 79 |
|  | Poultry drinking water | 20 | 7 | 138 | 2 | 15 | 19 | 2 | 49 |
|  | Cleaning poultry sewage | 25 | 58 | 106 | 0 | 10 | 15 | 3 | 32 |
|  | Poultry chopping board surface | 28 | 37 | 76 | 1 | 11 | 6 | 1 | 24 |
|  | Others | 0 | 0 | 2 | 0 | 0 | 0 | 0 | 0 |
| Season | Spring | 57 | 125 | 221 | 1 | 15 | 50 | 5 | 81 |
|  | Summer | 13 | 30 | 154 | 2 | 4 | 26 | 1 | 54 |
|  | Autumn | 14 | 10 | 120 | 0 | 5 | 11 | 0 | 58 |
|  | Winter | 46 | 41 | 273 | 2 | 40 | 27 | 3 | 76 |
