# Supplementary material for: A study of the relationship between human infection with avian influenza a (H5N6) and environmental avian influenza viruses in Fujian, China
Source: BMC Infect Dis. 2019 Sep 2;19:762. doi: 10.1186/s12879-019-4145-6 (PMC6719373; doi:10.1186/s12879-019-4145-6)
Supplement: Supplementary file 2 — Table S2. The GISAID isolate ID of H5N6 viruses. (DOCX 16 kb) [file 12879_2019_4145_MOESM2_ESM.docx]

| Viral Strains | GISAID Isolate ID |
| --- | --- |
| A/Environment/Fujian/05324/2016 | EPI_ISL_333631 |
| A/Environment/Fujian/05326/2016 | EPI_ISL_333630 |
| A/Environment/Fujian/09991/2016 | EPI_ISL_333632 |
| A/Environment/Fujian/28681/2016 | EPI_ISL_333633 |
| A/Environment/Fujian/28686/2016 | EPI_ISL_333634 |
| A/Environment/Fujian/52356/2016 | EPI_ISL_333636 |
| A/Fujian-Sanyuan/21099/2017 | EPI_ISL_304404 |
| A/Environment/Fujiansanyuan/08/2017 | EPI_ISL_333629 |
| A/Anhui/33163/2016 | EPI_ISL_284651 |
| A/Hubei/29578/2016 | EPI_ISL_256213 |
| A/Guangdong/99710/2014 | EPI_ISL_219828 |
| A/Changsha/1/2014 | EPI_ISL_217025 |
| A/Guangdong/ZQ874/2015 | EPI_ISL_206569 |
| A/Shenzhen/1/2015 | EPI_ISL_205313 |
| A/Guangzhou/39715/2014 | EPI_ISL_175335 |
| A/Sichuan/26221/2014 | EPI_ISL_163493 |
