# Supplementary material for: A study of the relationship between human infection with avian influenza a (H5N6) and environmental avian influenza viruses in Fujian, China
Source: BMC Infect Dis. 2019 Sep 2;19:762. doi: 10.1186/s12879-019-4145-6 (PMC6719373; doi:10.1186/s12879-019-4145-6)
Supplement: Supplementary file 3 — Phylogenetic analysis of the H5N6 viruses isolated in Fujian Province. (In red Triangle) Viral strains of human infection with avian influenza A(H5N6) virus in Fujian Province. (In green Square) Viral strains of human H5N6 viruses. (In red Circle) Viral strains of H5N6 viruses isolated from environment sample in Fujian Province. (DOC 415 kb) [file 12879_2019_4145_MOESM3_ESM.doc]

**PB2**

**PB1**

**PA**

**HA**

**NP**

**NA**

**MP**

**NS**
